# Random Bin for Analyzing Neuron Spike Trains

**DOI:** 10.1155/2012/153496

**Published:** 2012-07-08

**Authors:** Shinichi Tamura, Tomomitsu Miyoshi, Hajime Sawai, Yuko Mizuno-Matsumoto

**Affiliations:** ^1^NBL Technovator Co., Ltd., 631 Shindachimakino, Sennan City, Osaka 590-0522, Japan; ^2^Department of Integrative Physiology, Graduate School of Medicine, Osaka University, Suita 565-0871, Japan; ^3^Graduate School of Applied Informatics, University of Hyogo, Kobe 650-0047, Japan

## Abstract

When analyzing neuron spike trains, it is always the problem of how to set the time bin. Bin width affects much to analyzed results of such as periodicity of the spike trains. Many approaches have been proposed to determine the bin setting. However, these bins are fixed through the analysis. In this paper, we propose a randomizing method of bin width and location instead of conventional fixed bin setting. This technique is applied to analyzing periodicity of interspike interval train. Also the sensitivity of the method is presented.

## 1. Introduction

Bin width setting is always a problem, since it affects largely analyzed results. Neural spike train usually has time-varying characteristics. Therefore, data length of spike train in stationary state with the same characteristics is often limited. That is, the number of stable data is limited, and therefore there exists limitation in decreasing bin width to analyze more precisely. The more troublesome problem is that the results become different by how much to set the bin width or even the initial position. 

 Bin size has been determined to optimize some performance measure of time histogram [1, 2], time precision [3–5], information [6], rate estimation [7], and so forth. However, their bins are fixed after being optimized/determined. To avoid such troublesome problem, binless analysis methods are also used [8–10].

 In this paper, we propose a method of setting various random bins. Random bin will be expected to decrease unfavorable effects up to the level of being neglectable. See the appendix section for preliminary easy explanation of the random bin.

## 2. Automutual Information of Spike-Interval Train

To analyze a spike train as a time sequence, there exist mainly 4 methods of (i) spectrum analysis [11] which includes sideband and therefore may be limited in precise time analysis, (ii) correlation [12] which reflects only linear relation, (iii) time histogram [1] whose precision may be limited by nonstationarity of the train, and (iv) information measure [6, 12, 13] which is expected to be possible to avoid such limitations. Automutual information method dealt in this paper belongs to (iv).

Mutual information (MI) is a measure of expressing common quantity of information between events *A* and *B*, as described by (1):

(1) $$\begin{array}{c} \text{MI}\left(A;B\right)=\sum_{A}^{}\sum_{B}^{}P\left(A,B\right)\log_{2}\left[\frac{P\left(A,B\right)}{P\left(A\right)\cdot P\left(B\right)}\right]\left[\text{bit}\right]. \end{array}$$

More specifically, this is the difference between joint probability *P*(*A*, *B*) and probability *P*(*A*) · *P*(*B*) in which *A* and *B* are assumed to be independent events. If *A* and *B* are indeed independent, they have no common information, and therefore the mutual information is zero. If we take an inter-spike interval train as *A*, and one shifted by *m* intervals as *B*, mutual information becomes automutual information (AMI).

## 3. Spike Train

**Figure 1:**
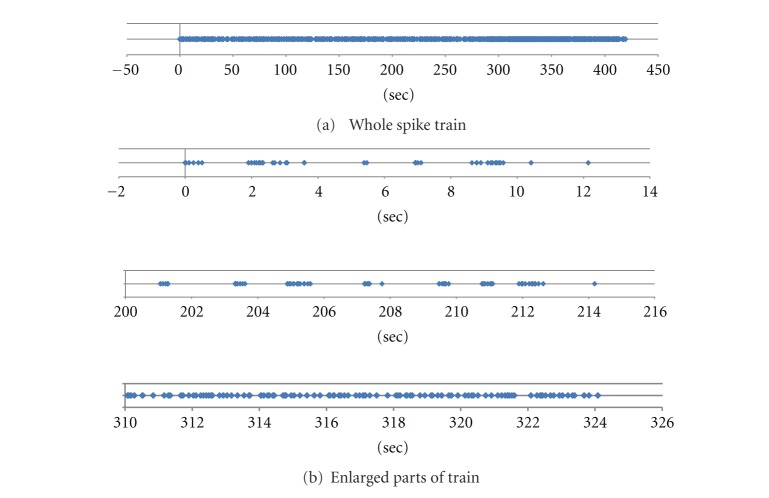
Example of sorted spike train (Electrode No. 16, light stimulated).

Figure 1 shows a spike train obtained from Electrode No. 16 of V1 field of a rat with LED light stimulation of 30 ms duration at every 7 sec. This is a sorted data, which means it is processed by pattern recognition so as to catch only spikes from a specific neuron. Number of spikes is 1721 between 420 sec. Some enlarged parts of the train are shown in Figure 1(b).

 To investigate the periodic characteristics, we calculated automutual information between interval-value train (*A*) and its shifted train (*B*) by *m* intervals.

## 4. Problem of Fixed Bin

**Figure 2:**
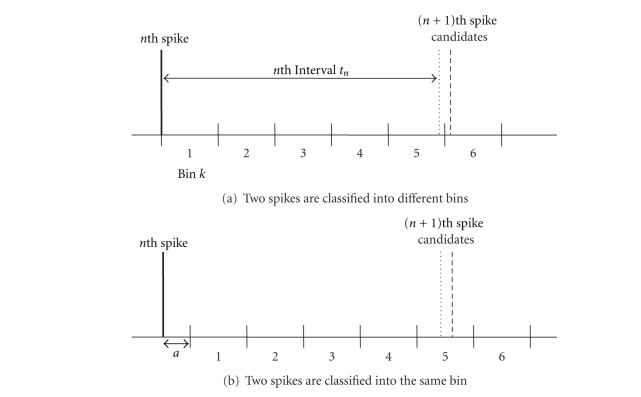
Problem of discriminatability in discretizing analog interspike intervals. Almost the same intervals are treated differently by chance in the fixed bin setting.

Figure 2 shows how almost the same intervals are classified into different bins or the same bin almost by chance depending on where the border of the bins is in the case of conventional fixed bin setting. This affects the result of AMI calculation. 

To show this, assume the bin is set as follows: number of bins is *K* = 32. The *k*th bin border (*k* = 1, 2, …, *K*) is given by

(2) $$\begin{array}{c} b\left(k\right)=0.01\times 10^{\left(3.5\times \left(k-a\right)/K\right)}=0.01\times 10^{\left(0.1094\times (k-a)\right)}. \end{array}$$

In this paper, to be able to cover wide range of intervals, exponential bin setting is adopted differently from Figure 2. The bin is set as

Bin 1: 0-b(1)
Bin 2: *b*(1)-*b*(2)
*⋯*
Bin 32: *b*(31)-*b*(32).

Data over *b*(32) were neglected. Usually we set *a* = 0 which corresponds to the case of Figure 2(a). In order to check the problem of the fixed bin in this section, we compared in cases of *a* = 0 and *a* = 0.5. That is, in the latter case, the Bin borders are shifted by half as Figure 2(b). This may often happen since spike interval has lower bound by refractory period, and therefore bin setting at small interval values is nonsense.

**Figure 3:**
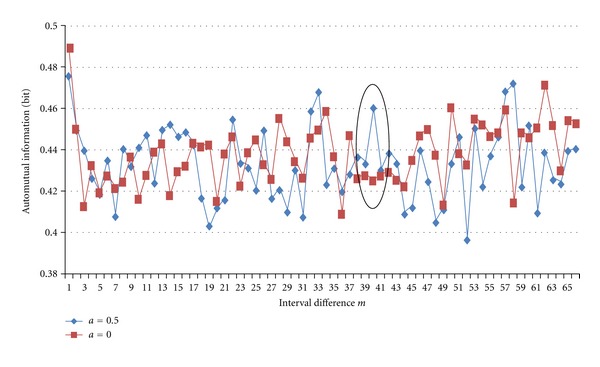
Difference of automutual information values for half-shift of bin position.

Figure 3 shows two results of AMI calculation for the data of Figure 1 with shifted bin positions by half as shown in Figure 2. Their shapes are rather different. For example, at *m* = 40 curve of *a* = 0.5 has a peak, but *a* = 0 has not been as shown by a black ellipse. We can see that it is almost impossible to extract period components from the spike train by the fixed bin setting as it is. 

## 5. Randomized Bin Setting

In order to suppress such instability, after having tried some methods including fluctuating initial position *a* of (2) and classifying an interval value into not one but adjacent two bins with weights, we decided finally to adopt a bin randomizing method, though it needs more computation time than the former. 

 First, 32 uniform random numbers between [0,32) are generated, rearranged in order from small to large, and they are substituted for *k* of (2). In a preliminary experiment, *K* was set 8, while it was set 32 in the main experiment, which was also extended to 128. Then we calculate one-trial AMI. Also start counting how many times one-trial AMI becomes the maximum at each *m* among, for example, 64 interval differences. This is a one trial with a random bin setting. We repeated these trials *N* = 5,000 to 500,000 times and averaged to obtain final AMI. At the same time, we also obtained normalized frequency of AMI becoming the maximum at each *m*. 

**Figure 4:**
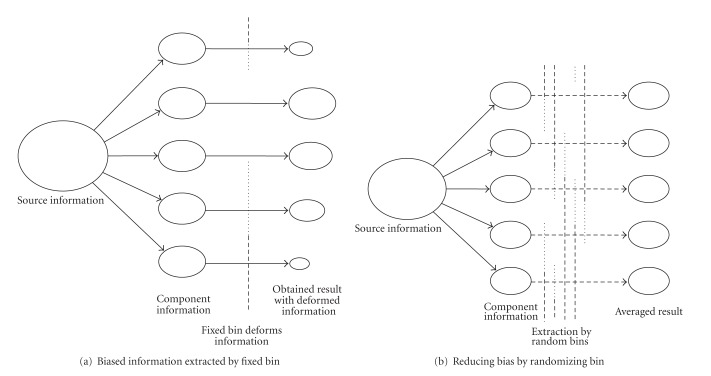
Reducing bias in obtained result by averaging randomly biased information.

This method will be explained in Figure 4; that is, fixed bin method (a) has some biased characteristics. If we generate random set of bin borders {*b*(*k*)} as method (b), bias effects will be decreased by repeating many times.

 In addition to the original data set (i) of spike trains from rat V1 field, we also prepared (ii) interval shuffled train (Shuf3/Shuf8) among successive 3 intervals or 8 intervals with sequentially shifting interval one by one and (iii) one repeated Shuf8 operation 2048 times (Shuf8–2048) or 256 times (Shuf8–256). Further we prepared (iv) three different randomly generated trains only having the same number of spikes with the original train but not the same interval distribution. 

## 6. Experimental Results

### 6.1. Preliminary Experiment

**Figure 5:**
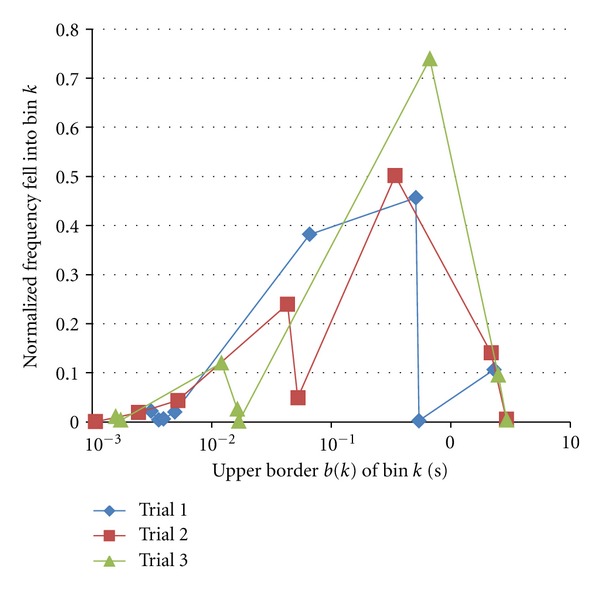
Random bin and rate fell into bin *k*. *K *= 8.

Before starting the main experiment, we tried with a small size of *K* = 8 and *N* = 5,000. Examples of normalized frequency that fell into bins in three trials for original train shown in Figure 1 are shown in Figure 5.

**Figure 6:**
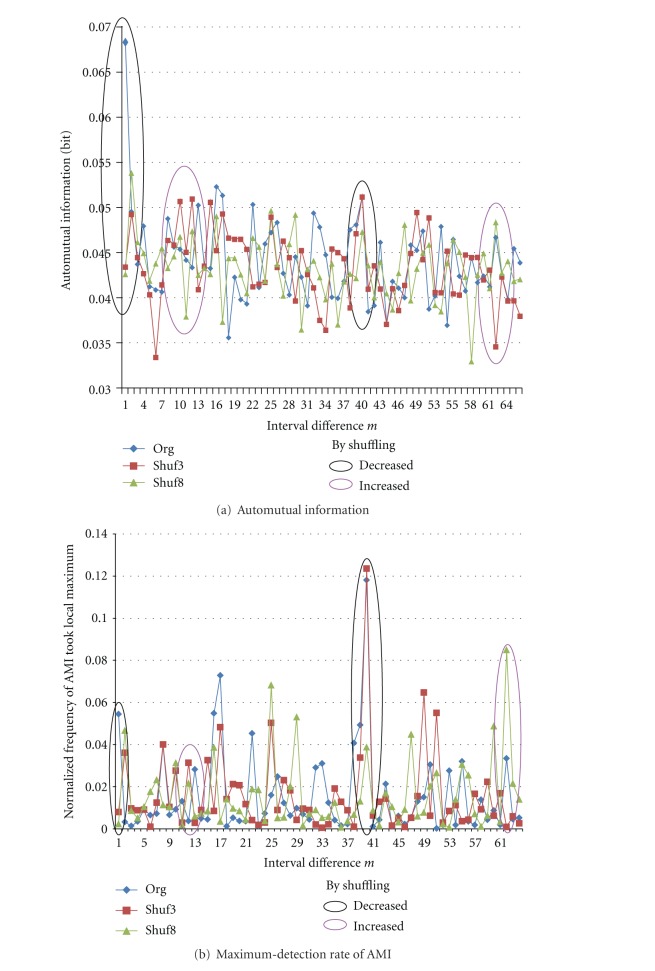
Changes by shuffling spike train for *K *= 8 and *N *= 5,000. In black ellipses as examples, AMI values and its peak detection rate are decreased according to the train shuffled more. On the other hand, in purple ellipses, they are increased by disturbance of shuffling.

Figure 6 shows changes of AMI and its frequency of taking local maximum at each interval difference *m* when shuffling the spike train. Generally speaking, by shuffling the train, AMI values do not decrease suddenly but gradually, since some rate of interval pairs moves in the same way with keeping the same relative interval difference. Large values of AMI and consequently large frequency of taking local maximum of Original train are often decreased by shuffling more as shown by black ellipses in Figure 6. Inversely, since total values of normalized frequency are 1, other new periodic components of AMI emerge/increase by shuffling, and consequently the rate of taking local maximum is also increased as shown by purple ellipses, though not completely. 

**Figure 7:**
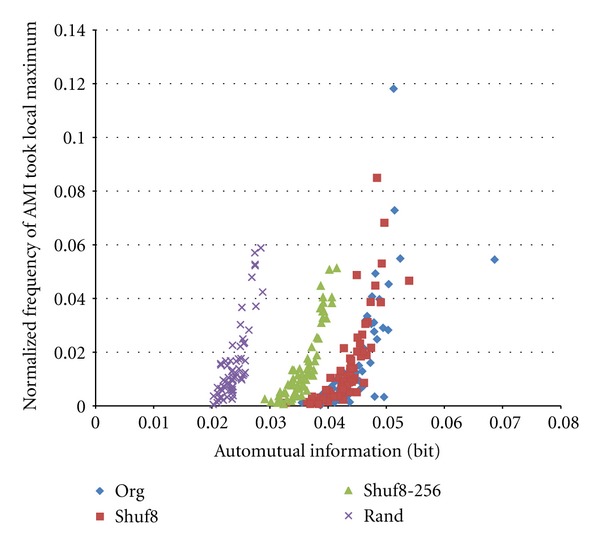
Scatter plot of automutual information versus probability of AMI that took local maximum. *K* = 8 and *N* = 5,000.

Figure 7 shows an obtained scatter plot of AMI versus frequency of AMI value took local maximum for original train shown in Figure 1, its Shuf8, Shuf8–256, and Random trains. AMI curve has such characteristics that (i) AMI of original train usually takes the maximum at *m* = 1, since if a spike detected that time moves to front, preceding interval value is shortened and succeeding one is elongated; that is they have negative correlation relationship (low independency), and (ii) curve is sometimes inclined subtly. To cope to these at this stage, we took a local-maximum judgment separately at ranges of 1–4, 5–8, 9–16, 17–32, and 33–64 instead of maximum judgment at full range of 1–64. Therefore we see 4 outlier points of Original data most at right and 3 around horizontal axis in Figure 7. We can also see that Shuf8 points are almost overlapping on the Original ones, Shuf8–256 points are shifted to lower AMI values, and Random points shifted more. These are well separated. That is, the AMI with random bin method can well extract temporal information of the spike train.

### 6.2. Prefinal Experiment

**Figure 8:**
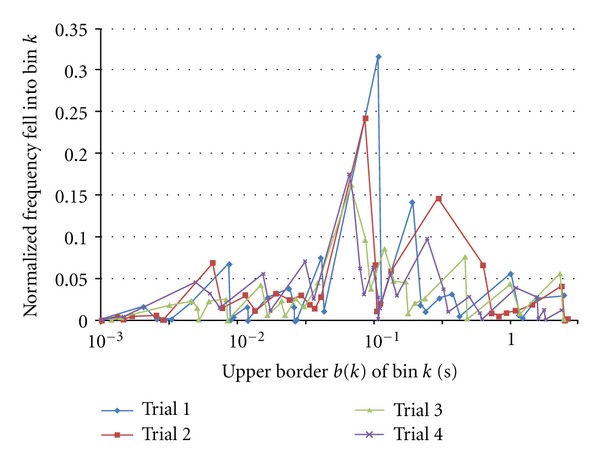
Examples of rate of intervals fell into bin *k*.

**Figure 9:**
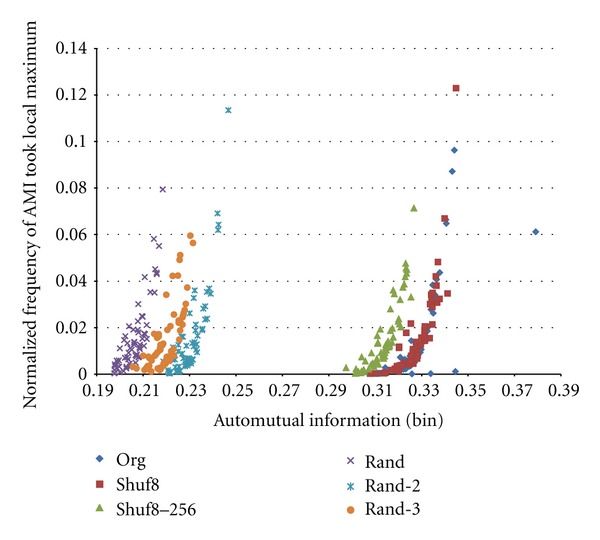
Scatter plot of automutual information versus probability of AMI that took local maximum.*K* = 32 and *N* = 40,000.

To improve the result, we expanded number of bins to *K* = 32 and number of trials to *N* = 40,000. When increasing *K* 4 times, since the probability of intervals falling into a bin decreases to 1/4, it may be reasonable also to increase *N* in this case 8 times. Examples of normalized frequency that fell into bins in four trials for original train shown in Figure 1 are shown in Figure 8.  Figure 9 shows scatter plot of the obtained AMI versus frequency of AMI value took the local maximum of the Original train shown in Figure 1 with its Shuf8, Shuf8–256 times, and three different Random trains. We can see that compared with Figure 7 the scatter plot converged more. Note that random trains have some divergence within trains.

### 6.3. Main Experiment

**Figure 10:**
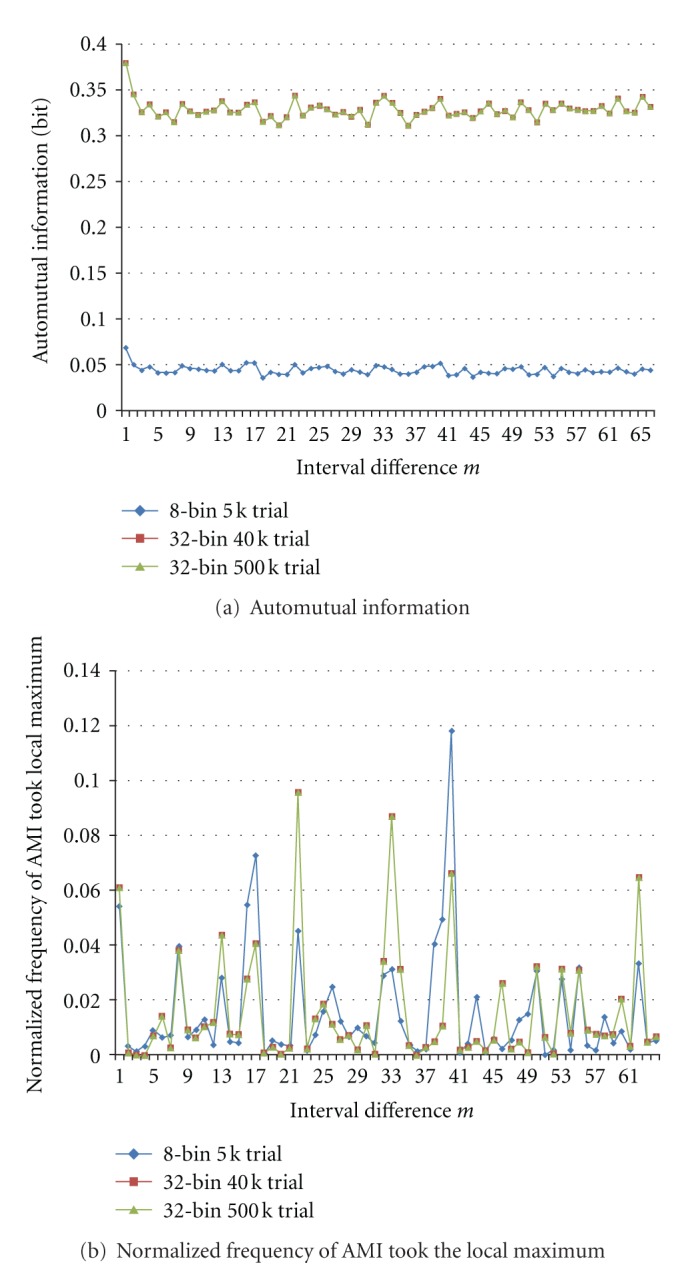
Improvement of AMI (automutual information) calculation and local maximum detection by increasing numbers of bins and trials. 32-Bin 40k Trial and 32-Bin 500 k curves are almost the same and overlapping.

**Figure 11:**
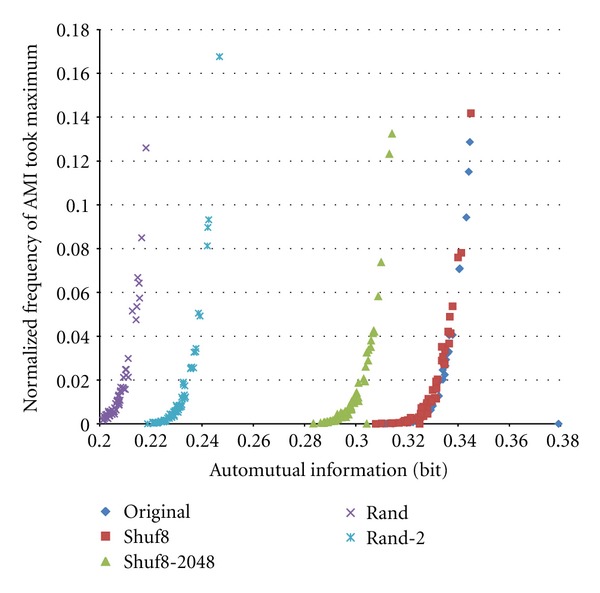
Scatter plot of automutual information versus probability of AMI that took the maximum for *K* = 32 and *N* = 500,000 with improving the maximum detection by suppressing AMI of *m* = 1. Electrode No. 16, light stimulated.

 Increasing the number of trials more to *N* = 500,000, we obtained almost the same results as *N* = 40,000 but more improved than *N* = 5,000, *K* = 8 (8Bins). These are shown in Figure 10. We can see that *N* seems to have reached plateau already at 40,000. In this case, by suppressing the AMI value at *m* = 1 to 0, we could determine more fairly the maximum of AMI value through all ranges of 64 interval differences. Then, we could obtain a final scatter plot of Figure 11, where we can see clear one-to-one correspondence between AMI and maximum-detection frequency than Figure 9.

 AMI curves in Figure 10(a) seem rather flat. Contrary to this, rate curve of AMI taking the maximum in Figure 10(b) appears more sensitive or too much sensitive to the periodicity. Essentially, however, they have the same information. 

## 7. Sensitivity Check

### 7.1. By Test Train Only

The average of inter-spike interval of the train No. 16 of Figure 1 was *τ* = (measuring time)/(number of spikes) = 0.244 sec. We generated a base train with constant interval of 0.244 sec. That is, the base train is

(3) $$\begin{array}{c} B=\left(t_{1},t_{2},\ldots ,t_{1721}\right)=\left(\tau ,\tau ,\ldots ,\tau \right). \end{array}$$

Then, test trains were generated by adding periodic component such that

(4) tn=(1+s)τ if  n=P×i,i=1,2,3,τ        otherwise,

where *P* is a period of the test component and *s* is its amplitude.

**Figure 12:**
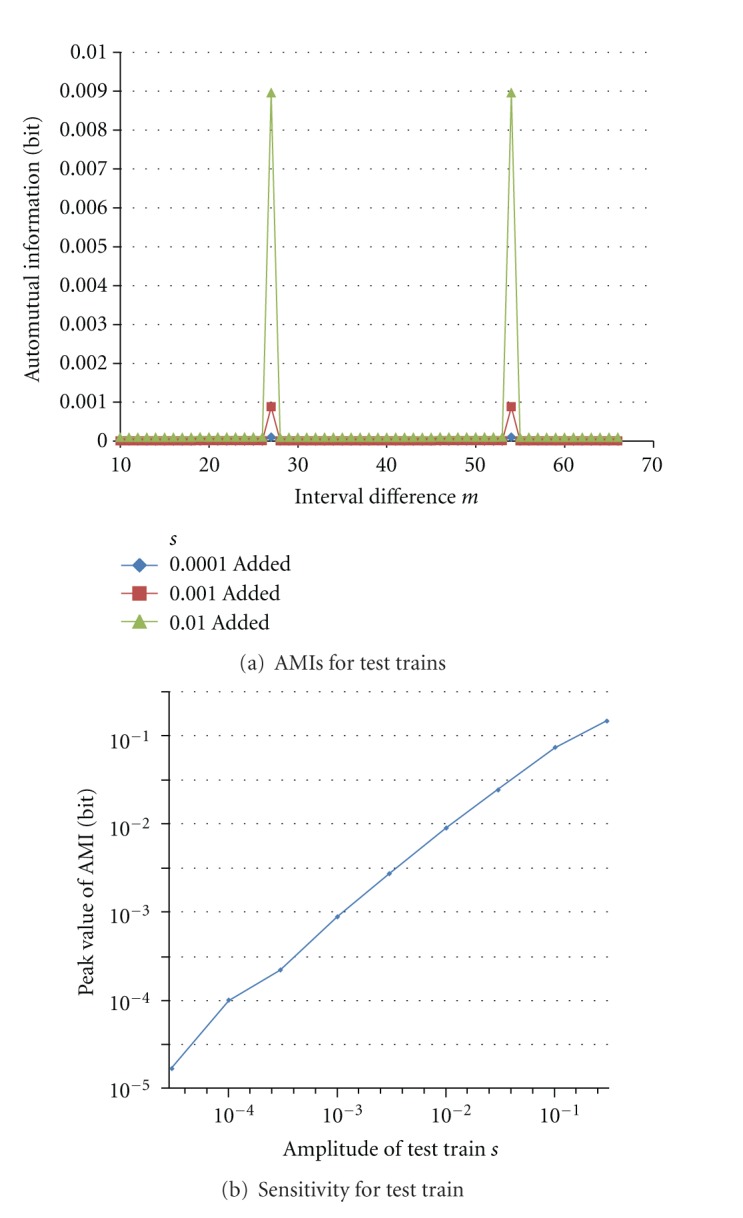
Sensitivity of AMI for various amplitudes of periodic test trains.

Figure 12(a) shows obtained AMIs for test trains with *P* = 27. It shows sharp peaks at *m* = 27, just corresponding to *P* as well as the 2nd peaks twice at interval difference *m* = 54.  Figure 12(b) shows their peak values at *m* = 27 with extending range of *s* more than (a). This is a sensitivity of the proposed method. 

### 7.2. Test Train Added to Real Train

**Figure 13:**
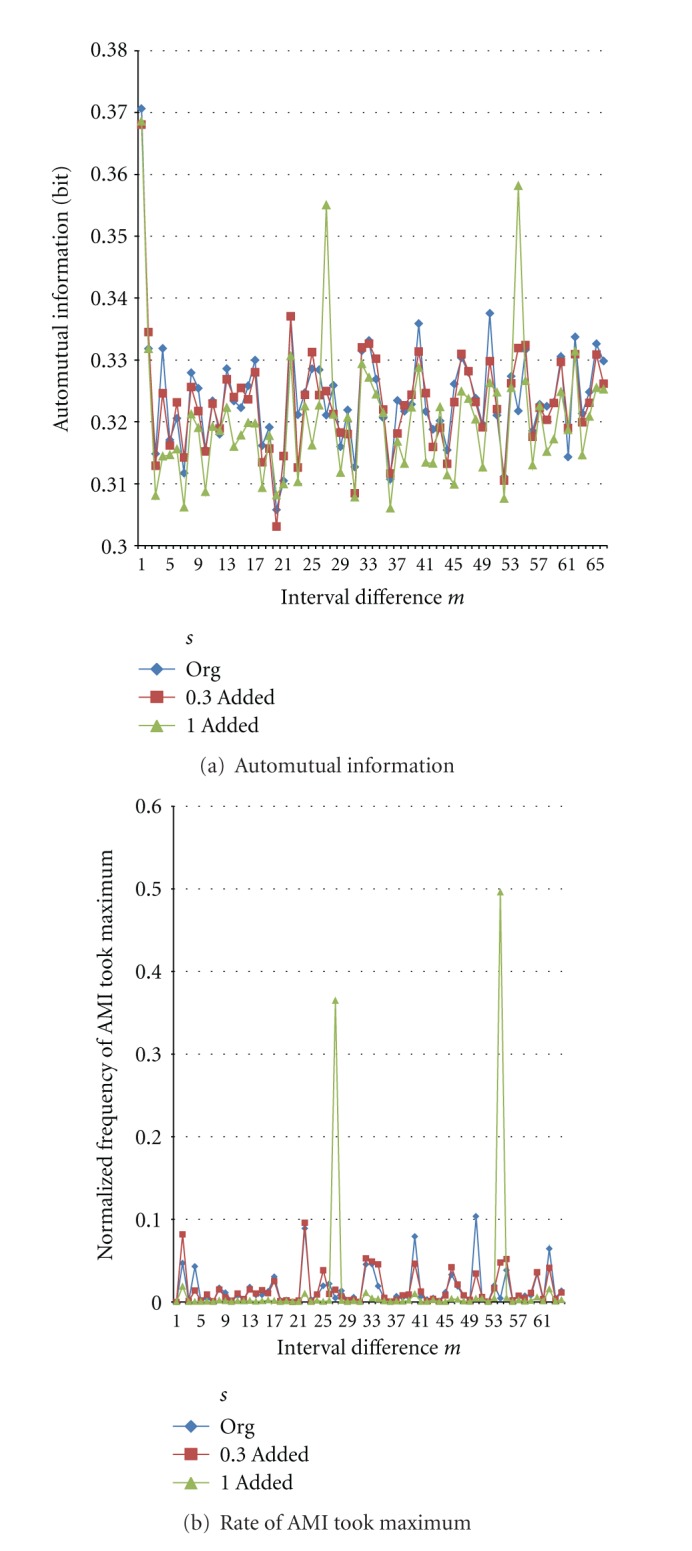
Results of test train added to No.16 train.

Figure 13 shows the results of test train with several amplitudes added to No.16 train. Test train is

(5) tn=sτ if  n=P×i  where  i=1,2,3,…0    otherwise,

where periodicity *P* = 27. We can see that in the Original train there exists low periodic component at *m* = 27. Then by adding test train with amplitude of more than 30% of average interval *τ*, periodic component appears; that is in No.16 train, there exist many periodic components with amplitude of several ten % of *τ*. 

## 8. Low Periodicity Train

**Figure 14:**
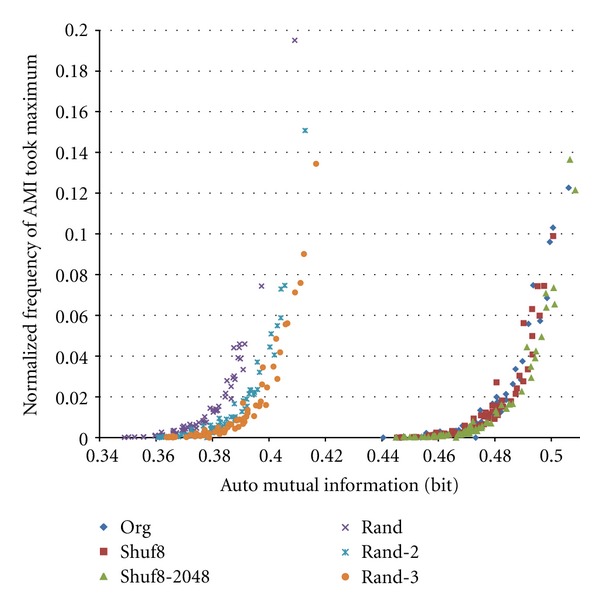
Example of typical scatter plot of common (ordinary) trains (nonstimulated; Electrode No. 2 sorted) of *K* = 32 and *N* = 40,000 with improved maximum detection.

The train from Electrode No. 16 with light stimulation shown above is the one mostly showing its deep structure in the sense that AMI values of Shuf8–2048 are clearly lower than that of Org. This means that characteristics including periodicity are disturbed by interval shuffling. However, this is not always the case. An example of results of commonly typical (ordinary) train of nonstimulated spontaneous response of No.2 Electrode is shown in Figure 14 where characteristics of shuffled train (Shuf8, Shuf8–256) are not so different from original one (Org) but have larger AMI values than that of artificially generated Random trains. Number of spikes in this No. 2 train is 729. 

## 9. Extension to 128 Bins

**Figure 15:**
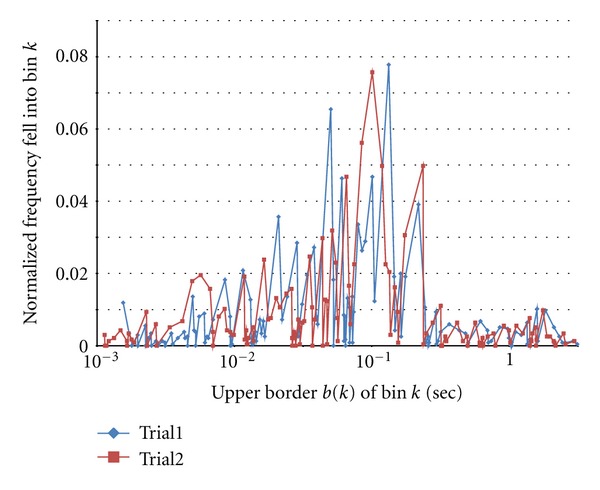
Two examples of rate of intervals fell into bin *k* when *K*=128.

**Figure 16:**
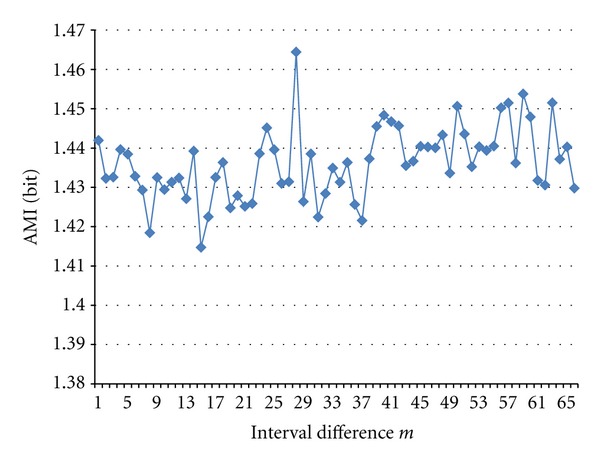
AMI of spike train No.2 with light stimulation and *K *= 128, *N *= 20,000.

**Figure 17:**
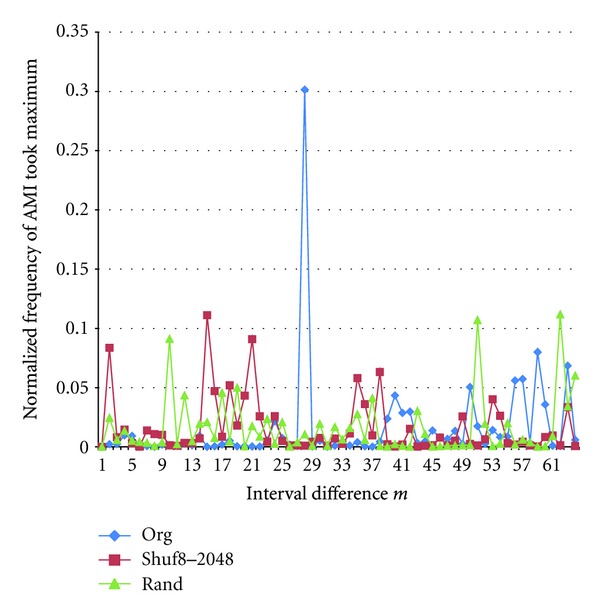
Normalized frequencies of AMI of Electrode No.2 with light stimulation took the maximum, interval shuffled (Shuf8-2048), and randomly generated (Rand).

We tried to extend the number of bins to 128 for some cases, though computation time takes several times compared with *K* = 32 cases.  Figure 15 shows two examples of rate of spike intervals fell into the random bins.  Figure 16 shows AMI, and Figure 17 shows the normalized frequency of AMI took the maximum of the spike train from the Electrode No. 2 with the light stimulations and *K* = 128, *N* = 20,000. We can see in this case that peak of AMI showing periodicity is sharp at *P* = 28, and it disappears after interval shuffling and in random sequence.

## 10. Discussion

**Figure 18:**
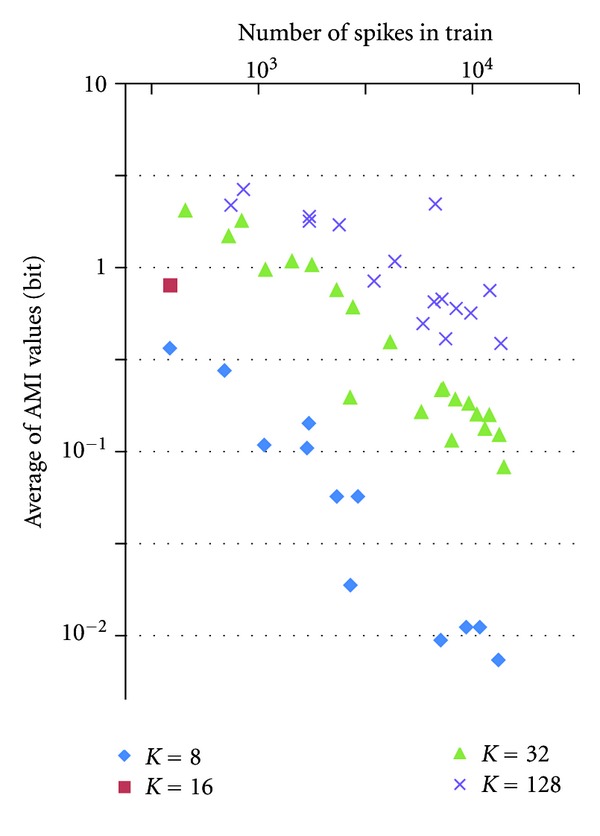
AMI level versus number of spikes. *N* = 5,000.

In the calculation of AMI, P(*A*,*B*) is estimated from the target data. As a result, it works as a learning effect. Consider an ultimate case with only two intervals *t*_1_ and *t*_2_ from three spikes, where we can estimate the future *t*_2_ (generally *t*_*n*+*m*_) perfectly with mutual information log_2_ *K* if we know *t*_1_(*t*_*n*_). Therefore, the smaller number of spikes we have, the more we can estimate future, and the higher mutual information we have between the present and future. Inversely, the larger number of spikes we have, the smaller level of the average AMI values we obtain.  Figure 18 shows the relation between AMI level and number of spikes in a train of our experiments. There may be some theoretical relationship between these. However, we have not analyzed enough yet. Instead in the experiment, we expanded *K*values up to 128 and can see that we can obtain higher AMI level which means we are able to estimate more accurate future interval values by increasing *K* value. However, it is also true that since the number of spikes is limited, we cannot increase *K* value unlimitedly to estimate well *P*(*A*,*B*).

From viewpoint of circuit theory, each periodicity corresponds to a specific circuit excited by a trigger input. Then, by analyzing the interspike interval sequence, it may be possible to get known the participating circuit shape or structure. Through such analysis, it may become possible to analyze the information storage and communication mechanisms in the brain.

 Problem of the proposed method is computation time. Presently software is written in Basic interpreter language (BASICw32), and it takes 8 hours with 2.4 GHz i5 CPU of note PC to calculate 40k trials when *K* = 32 for train data with 1721 intervals. This may be possible to reduce to one severalth by using compiler language.

## 11. Conclusion

Sizes and positions of time bins have been usually fixed. It often causes effect to precision and stability of the results. In this paper, we proposed a bin randomizing method to avoid such troubles. As an analyzing method, we used automutual information, which has merits of (i) detectability of even non-monotonic relation than correlation (ii) since the AMI is calculated based on not the absolute time but the appearance order and independence relationship between train intervals, it can cope with nonstationarity such as expand and contract of the spike interval combined with the flexibility of randomized bin, and it has more (iii) direct precise analysis than spectrum analysis and able to cope with nonstationarity. 

Demerit of the proposed method is the long computation time. However, as a postprocessing of the spike train, these are not severe demerits.

 It is shown that there exists an almost one-to-one monotonic relation between AMI value and rate of AMI value, takes the maximum through many trials of random bin generation. 

 Though mainly we treated a problem of obtaining automutual information, the proposed method of random bin can also apply not only to the spike analysis but also to other problems of other fields.

## Figures and Tables

**Figure 19:**
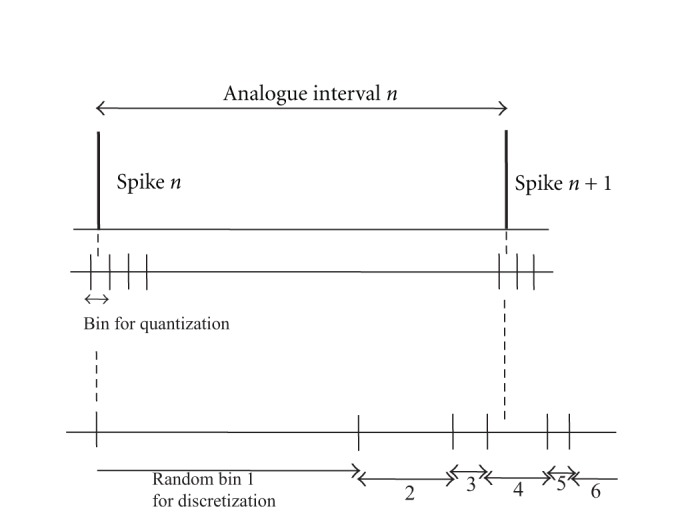
Conventional fixed bin for quantization (upper) and proposed random bin for time discretization (lower) to calculate mutual information. In this figure case, interval *n* is categorized to Random bin 4.

## Appendix

### Preliminary Explanation of Random Bin Proposing

If the codes for communication are generated repeatedly by circulating pulses in a loop circuit, we can observe a sequence with a period. Then two time intervals separated with some number *p* of intervals will differ in their lengths reflecting local appearance patterns in the code such as 11, 101,10 01, and 10001 as well as subtle physical transmission-time differences between different cell connections. Therefore, if we calculate mutual information between time lengths of the two intervals, we may be able to estimate the period of the code. Conventionally in this calculation, time bin size is first fixed, and analogue time lengths of intervals are next quantized according to this fixed bin size as shown in Figure 19 upper part. Typically bin size (width) is set to around time fluctuation (uncertainty) of spike position or small so as no more one spike falls in a bin. However, the bin size determined beforehand affects the results considerably. Therefore, we cannot trust any more the results obtained through this fixed bin size.

To cope with this problem, we propose here a method of discretizing analogue time intervals by random time-bins as shown in Figure 19 lower part for calculating the mutual information in each trial. These time bins are different trial by trial. Some time, the time bins are well determined and can discriminate different time intervals, and some time not. As an average, the mutual information will show the true mutual information not affected by the bin or each bin size.

Finally, the mutual information between interval *n* and interval *n* + *m* is shown as an automutual information (AMI) graph by changing *m* (interval difference).

## References

[1] Omi T, Shinomoto S (2011). Optimizing time histograms for non-Poissonian spike trains. *Neural Computation*.

[2] Shimazaki H, Shinomoto S (2007). A method for selecting the bin size of a time histogram. *Neural Computation*.

[3] Endres D, Oram M (2010). Feature extraction from spike trains with Bayesian binning: “Latency is where the signal starts“. *Journal of Computational Neuroscience*.

[4] Endres D, Schindelin J, Földiák Peter P, Oram MW (2010). Modelling spike trains and extracting response latency with Bayesian binning. *Journal of Physiology Paris*.

[5] Louis S, Borgelt C, Grün S (2010). Complexity distribution as a measure for assembly size and temporal precision. *Neural Networks*.

[6] Alan DD (2011). Estimating neuronal information: logarithmic binning of neuronal inter-spike intervals. *Entropy*.

[7] Shimazaki H, Shinomoto S (2010). Kernel bandwidth optimization in spike rate estimation. *Journal of Computational Neuroscience*.

[8] Paiva ARC, Park I, Príncipe JC (2010). A comparison of binless spike train measures. *Neural Computing and Applications*.

[9] Kruskal PB, Stanis JJ, McNaughton BL, Thomas PJ (2007). A binless correlation measure reduces the variability of memory reactivation estimates. *Statistics in Medicine*.

[10] Schrauwen B, Campenhout JV (2007). Linking non-binned spike train kernels to several existing spike train metrics. *Neurocomputing*.

[11] Rivlin-Etzion M, Ritov Y, Heimer G, Bergman H, Bar-Gad I (2006). Local shuffling of spike trains boosts the accuracy of spike train spectral analysis. *Journal of Neurophysiology*.

[12] Scaglione A, Foffani G, Scannella G, Cerutti S, Moxon KA (2008). Mutual information expansion for studying the role of correlations in population codes: how important are autocorrelations?. *Neural Computation*.

[13] Ito S, Hansen ME, Heiland R, Lumsdaine A, Litke AM, Beggs JM (2011). Extending transfer entropy improves identification of effective connectivity in a spiking cortical network model. *PLoS ONE*.

